# Cranberry, but not D-mannose and ibuprofen, prevents against uropathogenic *Escherichia coli*-induced cell damage and cell death in MDCK cells

**DOI:** 10.3389/fmicb.2023.1319785

**Published:** 2023-11-30

**Authors:** Jenane Konesan, Jenny Wang, Kate H. Moore, Kylie J. Mansfield, Lu Liu

**Affiliations:** ^1^School of Biomedical Sciences, UNSW Sydney, Sydney, NSW, Australia; ^2^St George Hospital, UNSW Sydney, Sydney, NSW, Australia; ^3^Graduate School of Medicine, University of Wollongong, Wollongong, NSW, Australia

**Keywords:** urinary tract infection, uropathogenic *Escherichia coli*, cranberry, D-mannose, ibuprofen, MDCK

## Abstract

**Introduction:**

The main function of the urinary tract is to form an impermeable barrier against urinary solutes and bacteria. However, this barrier can be compromised by urinary tract infections, most commonly caused by uropathogenic *Escherichia coli* (UPEC). This can result in damage to the epithelial barrier, leading to decreased epithelial thickness, loss of tight junctions, loss of epithelial integrity, and apoptosis. Due to the rise in antimicrobial resistance, there is worldwide interest in exploring non-antibiotic agents as alternative therapy.

**Methods:**

Using the Madin-Darby canine kidney (MDCK) cell line, a widely accepted epithelial cell model for the urinary tract, and the UPEC strain UTI89, this paper aimed to investigate the impact of UPEC on cell integrity, permeability, and barrier functions, and determine whether cranberry, D-mannose and ibuprofen could counteract the effects induced by UPEC. Furthermore, the study examined the protective potential of these agents against UPEC-induced increase in reactive oxygen species (ROS) production and programmed death-ligand 1 (PD-L1) expression.

**Results:**

The results demonstrated that UTI89 caused a marked reduction in cell viability and monolayer integrity. Cranberry (3 mg/mL) was protective against these changes. In addition, cranberry exhibited protective effects against UPEC-induced damage to cell barrier integrity, escalation of oxidative stress, and UPEC/TNFα-triggered PD-L1 expression. However, no effect was observed for D-mannose and ibuprofen in alleviating UPEC-induced cell damage and changes in ROS and PD-L1 levels.

**Conclusion:**

Overall, cranberry, but not D-mannose or ibuprofen, has a protective influence against UPEC associated damage in urinary epithelial cells.

## 1 Introduction

Urinary tract infections (UTIs) are among the most common infectious conditions worldwide. They affect more than 50% of women during their lifetime (Foxman, 2002). Infection of the urinary tract is commonly caused by gram-negative bacteria, particularly uropathogenic *Escherichia coli* (UPEC) (Flores-Mireles et al., 2015; Behzadi et al., 2023), which initiates approximately 80% of UTIs in women (Lee et al., 2011). These organisms infect and cause damage to the urinary bladder (Wood et al., 2012; Tian et al., 2018) and the kidney (Lane and Mobley, 2007), potentially disrupting the critical barrier functions of these tissues which usually function to prevent the reabsorption of excreted solutes (Acharya et al., 2004; Khandelwal et al., 2009).

UTIs arise from the adherence of UPEC to the urothelium or renal epithelium through surface appendages of type 1 or type P fimbriae (Gunther et al., 2001; Behzadi, 2020; Khonsari et al., 2021). Following binding, UPEC attempts to evade the host immune surveillance by being internalized and undergoing replication within the host cell (Obrien et al., 2016). Intracellular growth of UPEC consequently leads to cell death, which in turn disrupts barrier function due to loss of tight junctions in both zonula occludens-1 (ZO-1) and occluding proteins in the urinary bladder due to loss of urothelial integrity (Tian et al., 2018). The invasion of UPEC also triggers a proinflammatory response to UPEC cytotoxins such as lipopolysaccharide (LPS), making up the bacterial cell wall (Aguiniga et al., 2016). This leads to the release of cytokines such as IL-6, IL-8 and tumor necrotic factor-alpha (TNF-α), which are part of the innate immune response that helps eradicate the infection (Schilling et al., 2001; Zhang and An, 2007; Al Rushood et al., 2020).

TNF-α signaling is an important mediator of the bladder mucosal immune response to UPEC invasion (Yu et al., 2019). The release of TNF-α from infected tissue facilitates UPEC clearance by recruiting immune cells and stimulating the release of other cytokines (Schilling et al., 2001; Chen et al., 2013). However, prolonged tissue exposure to TNF-α has also been associated with elevated expression of programmed death-ligand 1 (PD-L1), an apoptotic marker that reduces the survivability and effector activity of T-cells (Hannan et al., 2014; Yu et al., 2019). PD-L1 overexpression is linked to poor disease elimination and recovery due to depleted T-cell responsiveness (Imai et al., 2020). Moreover, it is hypothesized that this cascade of inflammatory events will lead to hypersensitivity of the afferent signaling nerves in the urinary tract, such as C-fibers responsible for nociception (pain) and tissue damage perception, and Aδ fibers that sense bladder fullness (Brierley et al., 2020). Hence, this inflammatory response to tissue damage is thought to trigger the symptoms commonly associated with UTIs, including pain, frequency and urgency (Bono et al., 2023).

Typically, UTIs are effectively treated with antibiotics (Pietrucha-Dilanchian and Hooton, 2016). However, UTIs are becoming progressively more difficult to treat due to the significant rise in antibiotic resistance over the last few decades (Eisenreich et al., 2022). As a result, there is considerable interest in identifying other non-antibiotic alternatives to potentially minimize the usage of antibiotics. Several non-antibiotic agents have been studied in clinical trials for the prevention or treatment of UTIs (Sihra et al., 2018), with the most commonly investigated non-antibiotic agents being cranberry (Foxman et al., 2015; Vostalova et al., 2015; Asma et al., 2018), D-mannose (Kranjčec et al., 2014; Salinas-Casado et al., 2020; Lenger et al., 2023) and ibuprofen (Gágyor et al., 2015; Bleidorn et al., 2016; Moore et al., 2019). Cranberry (Gupta et al., 2007; Lavigne et al., 2008; Howell et al., 2010) and D-mannose (Schaeffer et al., 1980; Wellens et al., 2008) have been shown to inhibit the binding of UPEC to the urothelium but show variable efficacy in clinical trials evaluating their ability to prevent UTI (Konesan et al., 2022). In contrast, clinical trials of ibuprofen have indicated that these agents prevent the symptoms of UTI (especially those associated with inflammation) but have no efficacy in preventing UPEC infections (Konesan et al., 2022).

The aim of this study was to examine the effect of UPEC on Madin-Darby canine kidney (MDCK) cell integrity, permeability, and barrier functions, and then to evaluate the ability of non-antibiotic agents (cranberry, D-mannose, and ibuprofen) to prevent the UPEC induced changes. In addition, the protective effects of these agents on UPEC induced oxidative stress and inflammation induced PD-L1 expression was also examined. The hypothesis was that cranberry and D-mannose would be effective in blocking binding of UPEC to the epithelial surface and thus prevent the bacteria induced damage to the epithelial barrier function.

## 2 Materials and methods

### 2.1 MDCK cell culture

MDCK type I cell line (Cell Bank Australia, ECACC catalog no. 85011435) was chosen as the cell model for this study and was optimized for the purpose of investigating the effects of UTI on epithelial barrier functions. There are two sub-types of MDCK cells, designated as type I and II MDCK cells (Capellini et al., 2020). The MDCK type I cell has been shown to present very high transepithelial resistance (TEER) values (>4,000 Ωcm^2^), indicating a very strong formation of tight junctions (Barker and Simmons, 1981). In comparison, MDCK type II cells have been shown to display much lower TEER values (<300 Ωcm^2^), thus indicating a weaker ability to form tight junctions (Barker and Simmons, 1981). MDCK type I cells have also been established as a suitable *in vitro* method to detect bacterial virulence (Hirakata et al., 2000) and for determining the pathogenesis of various bacterial infections, including UPEC (Altman et al., 2001; Lin et al., 2006).

MDCK cells were cultured in Minimum Essential Medium (MEM), including 10% fetal bovine serum (FBS) and 1% penicillin–streptomycin (PS) in T75 flasks at 37°C in an incubator with 5% CO_2_ until they reached 70–80% confluence (approximately 4–5 days). At confluence, washed cells were passaged with Trypsin–EDTA (0.25%, 25,200,072, Thermo Fisher Scientific) for 5 min and then centrifuged at 750 g for 5 min. MDCK cells were then plated into T75 flasks or assay plates as appropriate.

### 2.2 Bacterial growth and cell treatments

**Figure 1:**
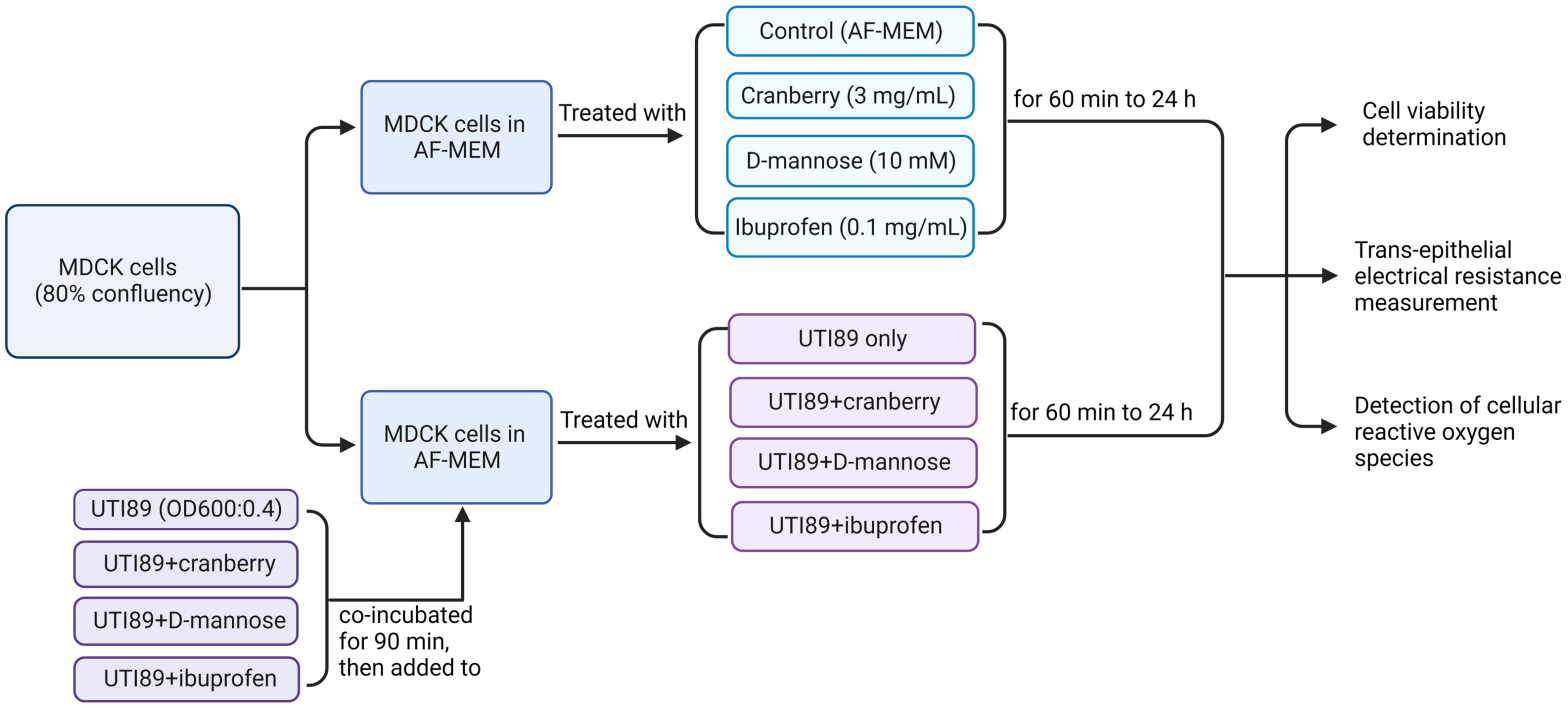
A flow chart of MDCK cells treated under various conditions to determine cell viability, trans-epithelial electrical resistance, and cellular reactive oxygen species. AF-MEM refers to antibiotic-free MEM. For UTI89 treatment groups, UTI89 (OD600: 0.4) was co-incubated with cranberry (3 mg/mL), D-mannose (10 mM), or ibuprofen (0.1 mg/mL) for 90 min at room temperature before being added to cultured cells.

A single bacterial colony of UTI89 isolate was resuspended in 10 mL of LB broth growth medium and grown overnight at 37°C. On the day of treatment, 1 mL of bacteria was resuspended in 9 mL LB broth and incubated at 37°C for another 1 h. Following the 1 h incubation, the bacteria were diluted to an optical density (OD600) of 0.4 (measured using Spectronic 20D+, ThermoFisher Scientific), equivalent to 2.0 × 10^8^ colony forming units. UTI89 was centrifuged at 5000 rcf (convert to g) and resuspended in equal amounts of antibiotic free MEM (AF-MEM), or AF-MEM containing cranberry, D-mannose or ibuprofen. UTI89 was preincubated with non-antibiotic agents for 90 min at room temperature before being used for cell treatments. MDCK cells were treated with these compounds for 1 h (Figure 1). Cranberry powder (Eclectic Institute 30,138) was dissolved in AF-MEM, supplemented with 10% FBS to a final concentration of 3 mg/mL (Gupta et al., 2007). The mixture was vortexed for 30 s and sterilized by filtration. D-mannose powder (Sigma M8574) was dissolved in AF-MEM with 10% FBS to a final concentration of 10 mM (Wellens et al., 2008) and sterilized through filtration. Ibuprofen was also dissolved in AF-MEM in 10% FBS to a final concentration of 0.1 mg/mL (Whiteside et al., 2019). It was noted that bacterial growth, as indicated by optical density (OD600), remained consistent across all conditions, whether UTI89 was cultured alone or in combination with cranberry, D-mannose, or ibuprofen, suggesting that these agents did not affect bacterial viability.

It is worth noting that prior to determining the optimal concentration, different concentrations of cranberry (1, 3, 10, and 30 mg/mL), D-mannose (1, 10, 30, and 100 mM), and ibuprofen (0.01, 0.1, and 1 mg/mL) were tested to measure trans-epithelial electrical resistance (TEER). A concentration of 3 mg/mL was chosen for cranberry due to its alignment with the linear curve (70% of the plateau response). Additionally, Gupta et al. (2007) also utilized this concentration to demonstrate cranberry’s inhibition of adherence of *E. coli* to cultured cells *in vitro*. The chosen concentrations ibuprofen (0.1 mg/mL) and for D-mannose (10 mM) for the subsequent experiments were consistent with published studies for ibuprofen (Whiteside et al., 2019) and D-mannose (Wellens et al., 2008), although a range of concentrations have been tested for both agents. D-mannose exceeding 30 mM demonstrated a toxic effect on the cell monolayer. Additionally, pre-incubation of MDCK cells with non-antibiotic agents before the addition of UTI89 was also evaluated. Consequently, the optimized conditions for the concentration of non-antibiotic agents and pre-incubation of UTI89 with non-antibiotic agents, as described above, were used for the experiments described.

### 2.3 Cell viability determination

To measure cell viability, MDCK cells (1 × 10^5^ cells per well) were plated in a 24-well plate (Corning, CLS3527-100EA) in MEM media (with 10% FCS and 1% penicillin streptomycin) and grown to 70–80% confluence (2–3 days). The day before the experiment, the complete MEM media was then aspirated and replaced with AF-MEM media. On the day of the experiment, the corresponding treatment was then added, including control (AF-MEM), cranberry (3 mg/mL), D-mannose (10 mM) or ibuprofen (0.1 mg/mL), with or without UTI89 (OD600: 0.4). Cell viability was measured using resazurin (10% of 0.3 mg/mL of stock, 199,303, Sigma-Aldrich). The baseline fluorescence signal was recorded by Flustar plate reader (560 nm excitation/590 nm emission). The plate was incubated at 37°C in 5% CO_2_ between readings and was read every 2 h over a total 6 h period. Photographs of the cells were taken at the end of each time point as well.

### 2.4 Trans-epithelial electrical resistance measurement

The protocol for measuring trans-epithelial electrical resistance (TEER) was adapted from the one used for TEER measurement in porcine urothelial cells (Taidi et al., 2022). In brief, once MDCK cells in T75 flasks had reached 80% confluence, they were passaged and plated (1 × 10^5^ cells per well) onto Corning Transwell inserts and cultured in MEM supplemented with 10% FBS and 1% penicillin streptomycin. TEER of MDCK cells was measured using the EVOM epithelial volt/ohm meter (EVOM, World precision instruments). The EVOM electrodes were sterilized with 70% ethanol and then rinsed with MEM culture media prior to each measurement. The short electrode was immersed in the apical compartment of Transwell inserts (containing the MDCK cells), while the long electrode was immersed in the basolateral compartment of the Transwell inserts (medium outside of the Transwell). TEER values were then monitored every other day until they reached at least 1,000 Ωcm^2^, which was achieved between 4 to 5 days. Complete MEM media was then aspirated and replaced with AF-MEM media a day before the experiment. On the day of the experiment, the corresponding treatment was then added, including control (antibiotic-free MEM media), cranberry (3 mg/mL), D-mannose (10 mM) or ibuprofen (0.1 mg/mL), with or without UTI89 (OD600: 0.4). TEER values were measured after 2, 4, 6 and 8 h.

### 2.5 Detection of cellular reactive oxygen species using 2′,7′- dichlorofluorescein diacetate assay

The level of cellular reactive oxygen species (ROS) was measured by 2′,7′-dichlorofluorescein diacetate assay (DCFH-DA). MDCK cells (5×10^4^ cells per well) were plated in a 96-well plate (black plate, clear bottom, Bio-strategy CORN3603) in MEM media (with 10% FCS and 1% penicillin streptomycin) at 37°C until confluence (approximately 2 to 3 days). Confluent MDCK cells were then treated with 2′,7′- dichlorofluorescein diacetate (DCFH-DA) dye (25 μM, Sigma D6883) in antibiotic-free MEM media with 10% FBS and incubated at 37°C in 5% CO_2_ for 45 min. The dye was aspirated, and the MDCK cells were washed with 200 μL of PBS (100 mM, pH 7.4). AF-MEM with 10% FCS was then added to the cultured wells. The cells were treated with UTI89 (OD600:0.4), with or without, cranberry (3 mg/mL), D-mannose (10 mM) or ibuprofen (0.1 mg/mL). The fluorescence was read using the FLUOstar Optima plate reader at an excitation/emission of 480/520 nm to establish the baseline reading. Plates were then incubated at 37°C in 5% CO_2_ and then the fluorescent signal was read at 2, 4, and 6 h to measure the ROS levels.

### 2.6 Determination of PD-L1 mRNA expression by real-time PCR

**Figure 2:**
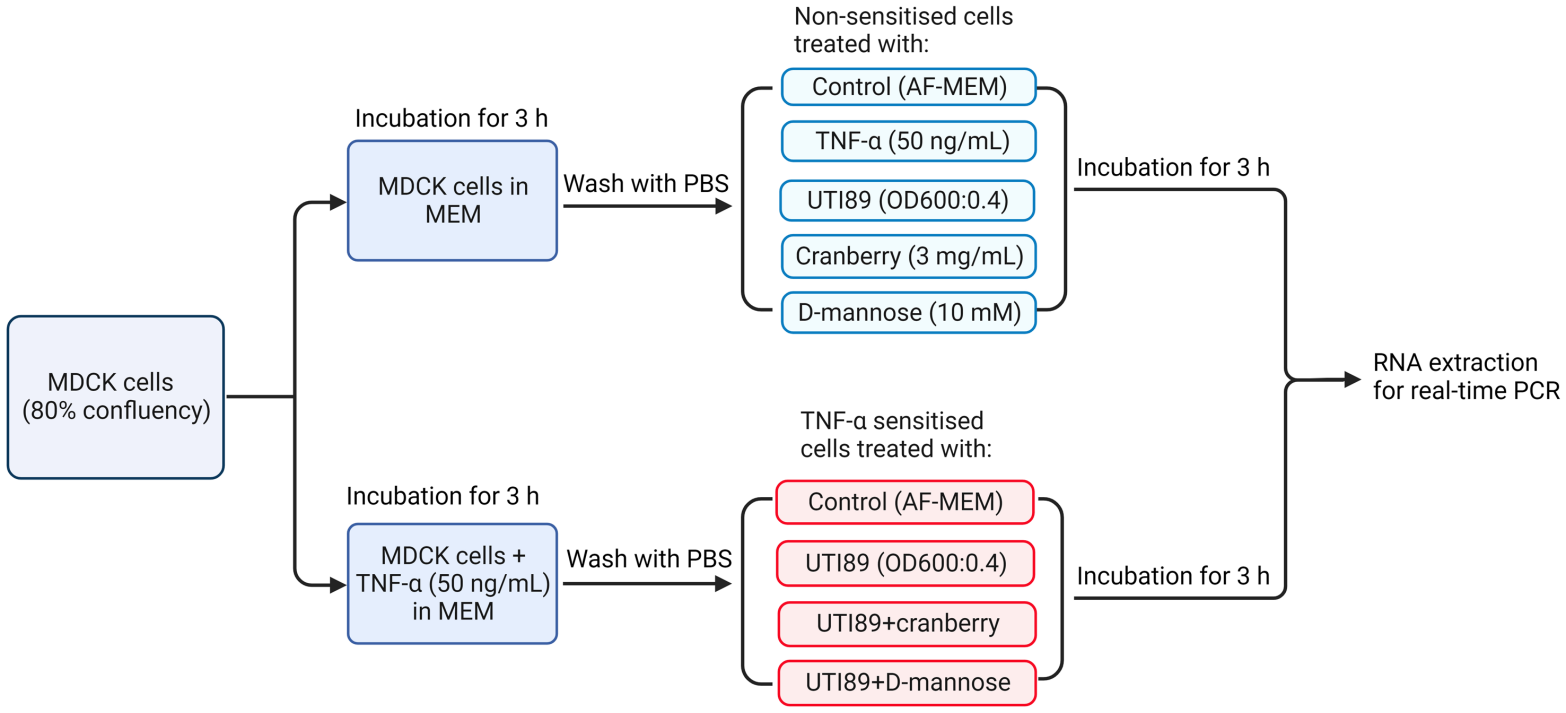
A flow chart illustrating the treatment of MDCK cells under various conditions, including non-TNF-α sensitized and TNF-α sensitized cells, for real-time PCR to determine PD-L1 mRNA expression. AF-MEM denotes antibiotic-free MEM. In the TNF-α sensitized groups, TNF-α (50 ng/mL) was added to cells cultured in complete MEM and incubated for 3 h. This was followed by washing with phosphate-buffered saline (PBS). For the UTI89 treatment groups, UTI89 (OD600: 0.4) was co-incubated with cranberry (3 mg/mL) or D-mannose (10 mM) for 90 min at room temperature before being added to the cultured cells.

MDCK cells were seeded into T25 culture flasks at 1 × 10^6^ cells each in complete MEM and cultured to 80% confluency. Human recombinant TNF-α solution (Abcam ab259410) was added to some cultures at a concentration of 50 ng/mL and incubated for 3 h. Subsequently, cell cultures were washed twice with PBS to remove antibiotics and dead cells, then treated with UTI89 (OD600 = 0.4) in the presence or absence of cranberry (3 mg/mL) or D-mannose (10 mM) for 3 h at 37°C in 5% CO_2_. Cells were then scrapped into media and resuspended in cold PBS (Figure 2).

Following centrifugation, cells were processed for total RNA extraction using TRIzol™ reagent (ThermoFisher Scientific 15,596,026) and the Precellys 24 Tissue Homogeniser (Bertin Instruments). RNA (2 μg) was converted to single-strand complementary DNA (sscDNA) using the Superscript™ III First-Strand Synthesis SuperMix (Thermofisher Scientific 18,080,051). Products of sscDNA were diluted to 500 ng/μL and subjected to real-time polymerase chain reaction (RT-PCR) using the KAPA SYBR FAST qPCR Kit (Kapa Biosystems KK4600). RT-PCR primer sequences were generated using Prism-3, based upon cDNA sequences for *Canis lupus familiaris* recorded in the NCBI GenBank database (the accession numbers for PD-L1 and GAPDH are NM_001291972 and NM_001003142, respectively). The primers for PD-L1 were CACGCTGAACATCAATGCAAC (forward) and CAACAGGAAAGGTCCCAGAA (reverse), and for GAPDH were AACTCCCTCAAGATTGTCAGC (forward) and GTGGAAGCAGGGATGATGTT (reverse). Amplicon lengths were confirmed by running agarose gel electrophoresis. RT-PCR was run in Eppendorf Mastercycler at 40 cycles, comprised of denaturation at 95°C 15 s, annealing at 60°C 15 s and extension at 68°C 20 s. The generated cycle threshold (Ct) values were used to quantify target gene expression as fold change (2^−ΔΔCt^), where ΔΔCt = [Ct_(target)_ – Ct_(GAPDH)_]sample – [Ct_(target)_ – Ct_(GAPDH)_] calibrator. Here, GAPDH was used as a housekeeping gene and a same sscDNA sample was run in each plate as an inter-run calibrator control.

### 2.7 PD-L1 immunofluorescence

MDCK cells were seeded onto 13 mm coverslips at 3.5 × 10^4^ cells each and cultured in complete MEM media. The cell treatment protocol closely followed the procedure outlined in Figure 2, except for extended incubation times. In brief, when cells reached 80% confluency, some cultures were sensitized with TNF-α (50 ng/mL) for an optimized 24-h duration. The media was aspirated, and the cells were washed with PBS and subjected to UTI89 (OD600:0.4) in the presence or absence of cranberry (3 mg/mL) or D-mannose (10 mM) for 6 h, at 37°C in 5% CO_2_. After incubation, cells were washed twice with PBS and fixed using 95% ethanol/5% acetic acid for 10 min at −20°C. To perform immunofluorescence, cells were washed with PBS and incubated with 10% donkey serum (Sigma 09663) in PBS for 45 min at room temperature. The primary antibody (Abcam ab205921, 1:100) was diluted in Tris-buffered saline with 0.05% Triton (TBS-TX) and 2% donkey serum. Cells were incubated with primary antibody overnight at room temperature, while the negative control was incubated in TBS-TX buffer only. Following primary antibody exposure, cells were washed 3 × 10 min with TBS and incubated with secondary antibody in the dark for 2 h at room temperature. The secondary antibody used was the Donkey anti-Rabbit IgG Alexa Fluor™ 568 (Abcam ab176692, 1:200), which was also diluted in TBS-TX. The cells were rinsed again 3 × 10 min in TBS, and the coverslips were mounted “face down” onto glass slides with DAPI media. Fluorescent images of the slides were examined with the Olympus BX51TF microscope, captured at ×40 magnification using Neurolucida software and superimposed with Image J software.

### 2.8 Statistical analysis

All data were analyzed using GraphPad Prism 9 and presented as the mean ± standard error of the mean (SEM) of three or more standalone experiments. Multiple group comparisons and grouped data for TEER, cell viability, ROS and PCR results were subjected to one-way or two-way ANOVA, followed by Bonferroni’s multiple comparisons test. Statistical significance was determined by a *p* value of <0.05.

## 3 Results

### 3.1 Effect of UPEC on MDCK cell morphology

**Figure 3:**
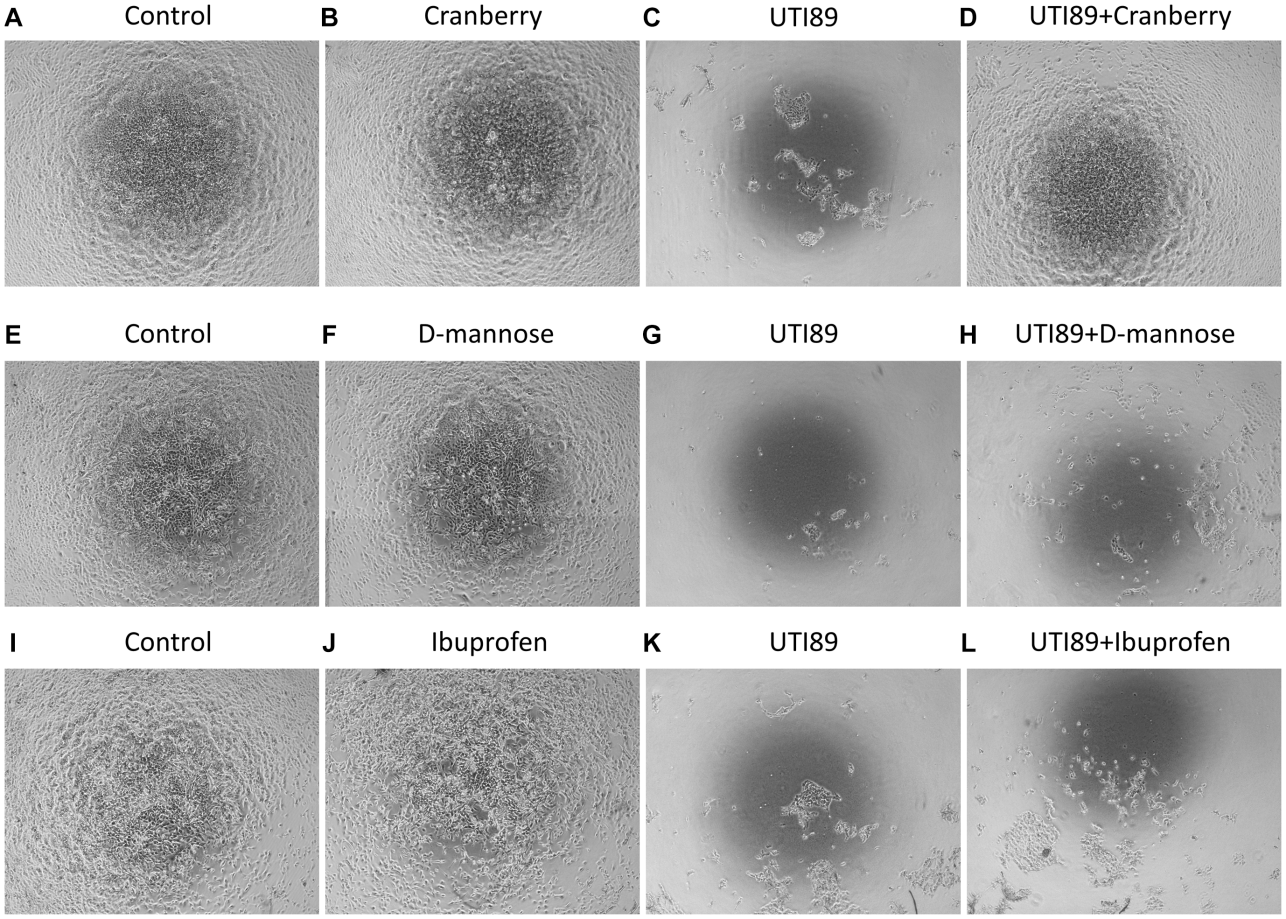
MDCK cell morphology under different treatments. Cells were photographed after 4 h treatments. Confluent MDCK cells under the control condition, incubated with antibiotic-free MEM, maintained the cell monolayer **(A,E,I)**. Cells treated with cranberry [3 mg/mL, **(B)**], D-mannose [10 mM, **(F)**] or ibuprofen [0.1 mg/mL, **(J)**] alone also showed complete preservation of the cell monolayer. Cells treated with UTI89 (OD600: 0.4) demonstrated significant disruption and loss of MDCK cells **(C,G,K)**. Co-incubation of cranberry with UTI89 exhibited a protective effect against UTI89-induced disruption **(D)**. However, this protective effect was not observed for D-mannose **(H)** or ibuprofen **(L)**.

MDCK cells incubated with AF-MEM (control, Figures 3A,E,I), cranberry (Figure 3B), D-mannose (Figure 3F) or ibuprofen (Figure 3J) for 4 h showed a well-attached, confluent cell monolayer. However, incubation with UTI89 for the same duration induced significant damage to the cell monolayer (Figures 3C,G,K). Interestingly, when UTI89 was co-incubated with cranberry, inhibition of damage to the cell integrity was observed, indicating a potential preventive effect of cranberry against bacterial-induced cellular damage (Figure 3D). In contrast, this protective effect was not observed for D-mannose (Figure 3H) or ibuprofen (Figure 3L) under the same conditions. These findings were consistent when the treatments were extended to 24 h (data not shown).

### 3.2 Effect of cranberry, D-mannose, and ibuprofen on UPEC-induced cytotoxicity

**Figure 4:**
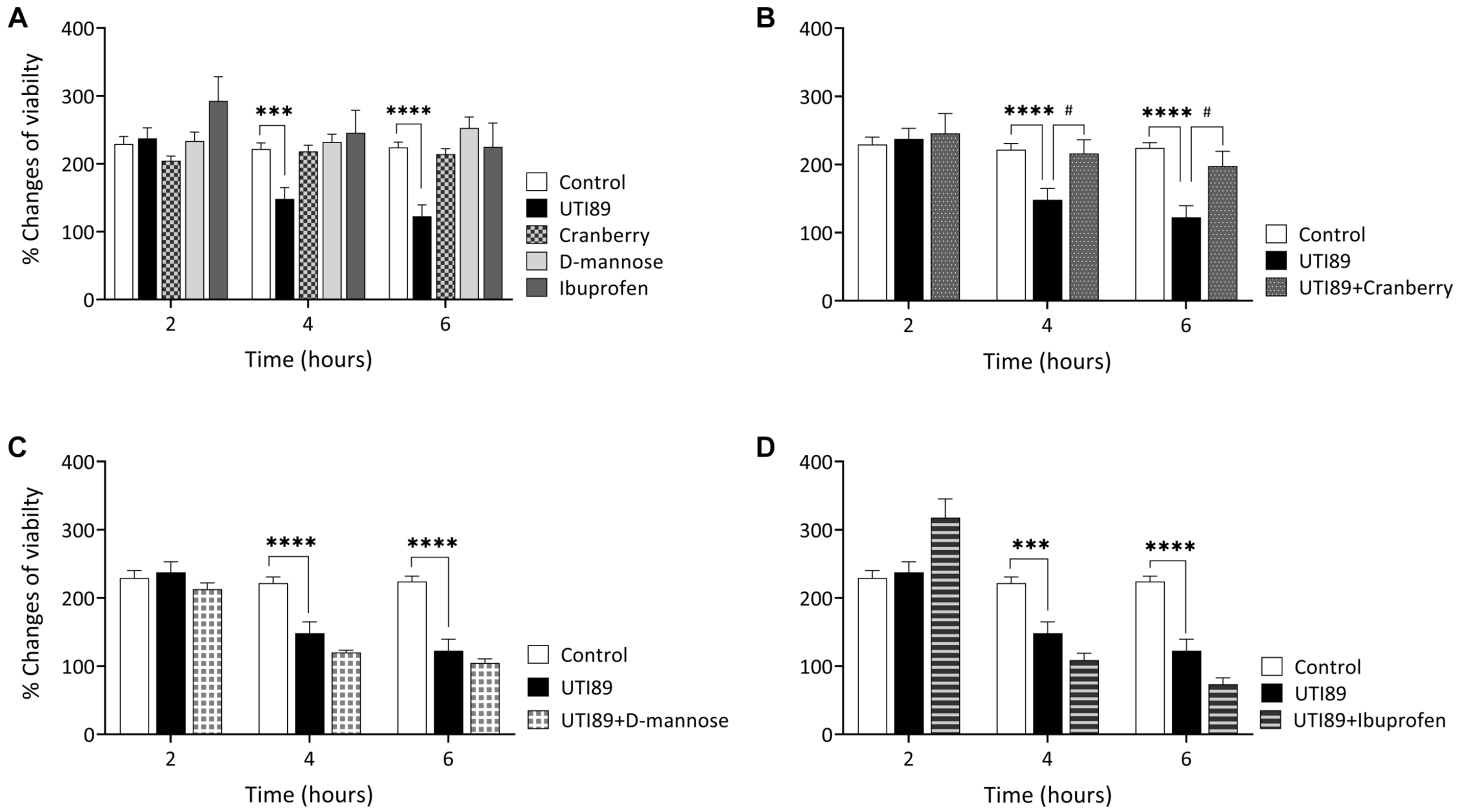
Effect of UTI on MDCK cell viability measured by oxidation of resazurin. Panel **(A)** shows that the control, cranberry, D-mannose, and ibuprofen, when applied alone, maintained consistent cell viability throughout the entire 6 h period. However, UTI89 significantly reduced cell viability at both 4 h (***p = 0.0005) and 6 h (****p < 0.0001), but not at 2 h, when compared to the control. Panel **(B)** demonstrates the protective effect of cranberry against UTI89, resulting in improved cell viability at 4 h (^#^*p* = 0.0295) and 6 h (^#^*p* = 0.0137), compared to the UTI89 treated group. However, panel **(C,D)** show that D-mannose and ibuprofen did not produce a protective effect against UTI89-induced cytotoxicity. The data were based on 6 replicates (*n* = 6), and statistical analysis was conducted using two-way ANOVA, followed by Bonferroni’s comparisons test.

UTI89 displayed a time-dependent reduction in cell viability. There was no observable effect of UTI89 after incubation for 2 h, however, increased cytotoxic effects were evident at later time points, as demonstrated by decreased cell viability at 4 and 6 h (*p* < 0.001 or *p* < 0.0001, Figure 4A). The application of cranberry (3 mg/mL), D-mannose (10 mM), and ibuprofen (0.1 mg/mL) alone did not exert any impact on cell viability at any of the time points (Figure 4A). When UTI89 was co-incubated with cranberry, the expected reduction in cell viability was not observed (*p* < 0.05 compared to UTI89 treatment, Figure 4B). However, this protective effect was not observed with D-mannose (Figure 4C) or ibuprofen (Figure 4D).

### 3.3 Effect of cranberry, D-mannose, and ibuprofen on UPEC-induced disruption to epithelial cell barrier function

**Figure 5:**
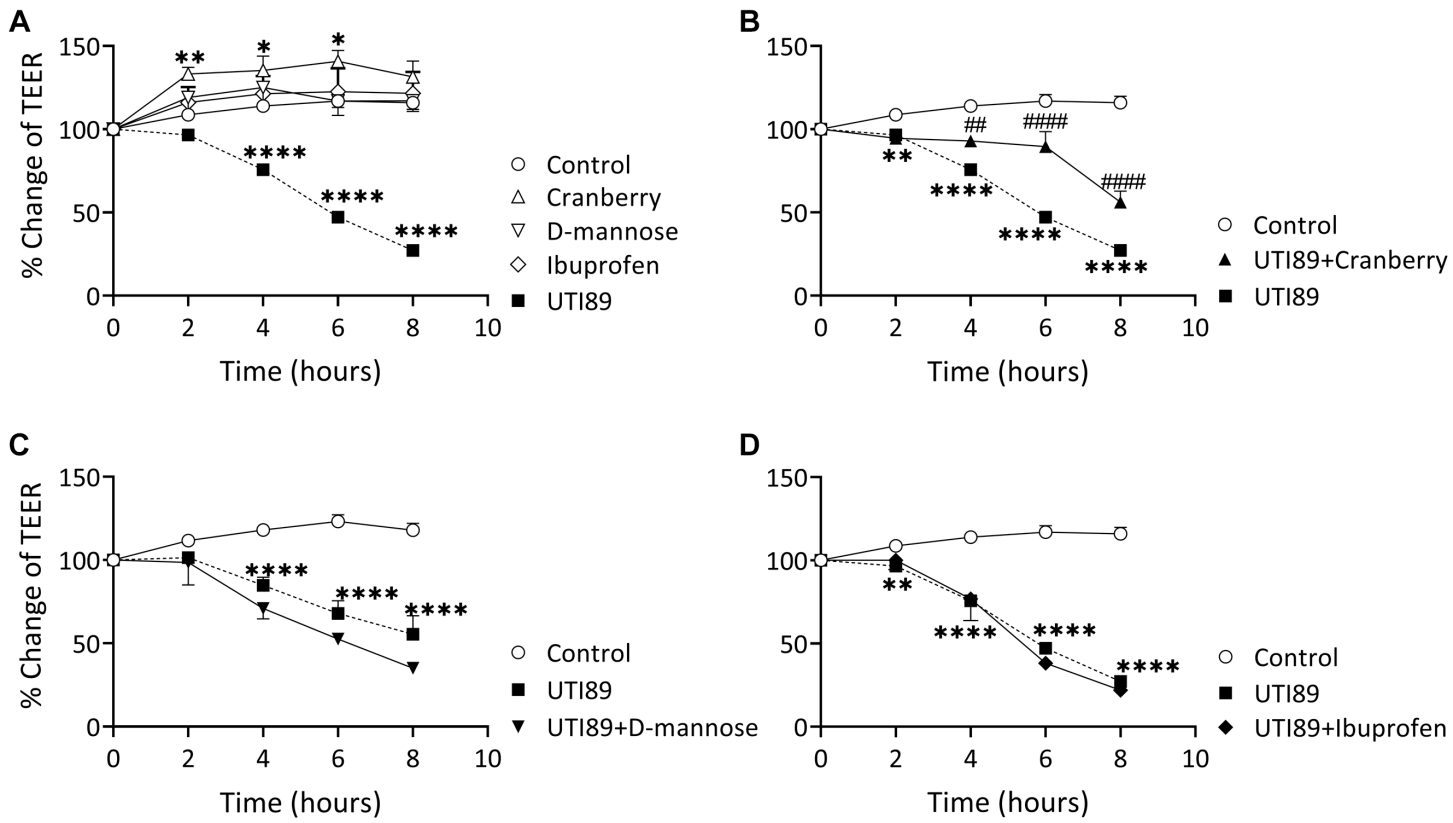
Effect of UTI89 on MDCK cell integrity measured by trans-epithelial cell resistance (TEER). **(A)** Shows that MDCK cells incubated with UTI89 demonstrated a significant reduction in TEER values at 4–8 h. TEER values in the cells treated with cranberry (3 mg/mL) displayed a small but significant increase at 2, 4, and 6 h. However, D-mannose (10 mM) or ibuprofen (0.1 mg/mL) remained stable over the entire 8 h period. **(B)** UTI89 incubated with cranberry partially blocked UTI89 caused reduction of TEER at 4 to 6 h. This protective action was not observed with **(C)**. D-mannose nor **(D)** ibuprofen when incubated with UTI89 over the entire 8 h period. The data were based on *n* = 5 individual experiments, and statistical analysis was conducted using two-way ANOVA, followed by Bonferroni’s comparisons test., *p < 0.05, **p < 0.01, ****p < 0.0001, compared to the control; ^##^*p* < 0.05, ^####^*p* < 0.0001, compared to the UTI89 treated group.

Incubation of MDCK cells with UTI89 (OD600: 0.4) induced a significant disruption to the barrier function of the epithelial cell monolayers, as evidenced through significant reductions in TEER at 4, 6, and 8 h in comparison to the control group treated with AF-MEM (*p* < 0.0001, as depicted in Figure 5A). The TEER values were reduced by as much as 60% at the 8 h mark. Interestingly, cells treated with cranberry (3 mg/mL) only, showed a slight but significant increase in TEER. On the other hand, cells treated with D-mannose (10 mM) or ibuprofen (0.1 mg/mL) did not show any changes in TEER values when compared to the control (Figure 5A). When cranberry was co-incubated with UTI89, it exhibited a protective effect against UTI89-induced TEER reduction at the 4, 6 and 8 h time points (Figure 5B, *p* < 0.01 compared to the UTI89 only group). However, a similar protective effect was not observed with D-mannose (Figure 5C) or with ibuprofen (Figure 5D) when incubated with UTI89 over the entire 8 h duration.

### 3.4 Effect of cranberry, D-mannose, and ibuprofen on UPEC-induced ROS production

**Figure 6:**
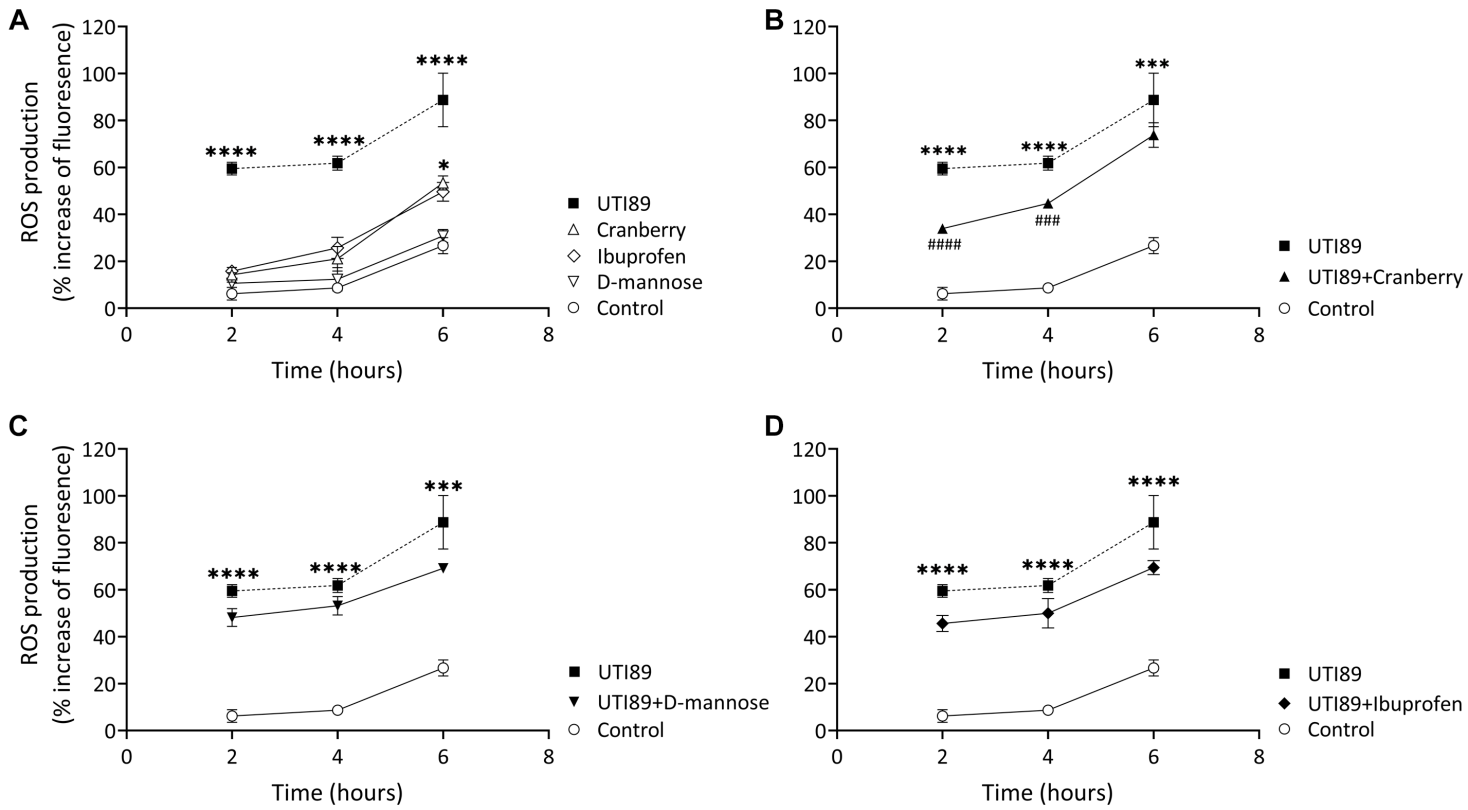
DCFH-DA assay to detect cellular ROS level. **(A)** While all treatment groups exhibited a time-dependent increase in ROS production, MCDK cells incubated with cranberry (3 mg/mL), D-mannose (10 mM) and ibuprofen (0.1 mg/mL) showed no significant difference compared to the control group, except for cranberry, which displayed a higher ROS level at 6 h. UTI89 (OD600: 0.4) significantly enhanced ROS levels at all time points. **(B)** Cranberry exerted a partial protective effect on UTI89-induced ROS production at 2 and 4 h. **(C)** D-mannose and **(D)** Ibuprofen did not demonstrate a protective effect against UTI89-induced ROS production. The data were collected from *n* = 4–12 individual experiments, and statistical analysis was conducted using two-way ANOVA, followed by Bonferroni’s comparisons test. *p < 0.05, ***p < 0.001, and ****p < 0.0001, compared to the control. ^###^*p* < 0.001, ^####^*p* < 0.0001 compared to the UTI89 treated group.

In all groups, there was an observable, time-dependent increase in ROS production (Figure 6A). ROS production was higher in cells incubated with UTI89 alone than in the control at all time points (*p* < 0.0001) (Figure 6A). Cranberry treatment reduced the UTI89-induced ROS production at 2 h (*p* < 0.0001) and 4 h (*p* < 0.001), although this effect was not maintained at 6 h (Figure 6B). Neither D-mannose (Figure 6C) nor ibuprofen (Figure 6D) was able to effectively lower UTI89-induced ROS production over the entire 6 h period.

### 3.5 Impact of UTI89 on PD-L1 mRNA expression and PD-LI immunoreactivity

**Figure 7:**
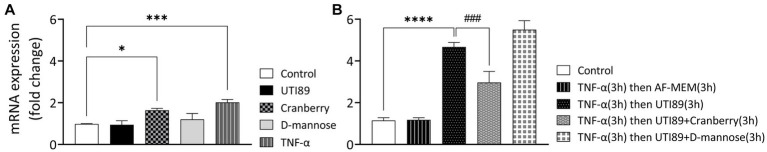
The effect of cranberry (3 mg/mL) and D-mannose (10 mM) on PD-L1 mRNA expression in MDCK cells. Results were obtained through real-time PCR analysis of *n* = 3–13 experiments. Separate controls were established for the two total durations of treatment [3 h panel **(A)** or 6 h panel **(B)**]. PD-LI mRNA expression levels were presented as mean ± SEM of fold changes, normalized to the housekeep gene GAPDH and an inter-run calibrator. All data were analyzed for significance using one-way ANOVA with Bonferroni’s multiple comparisons test. *p < 0.05; ***p < 0.001; ****p < 0.0001 compared to corresponding untreated control. ^###^*p* < 0.001 compared to indicated non-control group.

Neither UTI89 (OD600: 0.4) nor D-mannose (10 mg/mL) alone induced any considerable change in PD-L1 mRNA expression after 3 h (Figure 7A). However, cranberry treatment (3 mg/mL) and TNF-α (50 ng/mL) led to a slight increase in PD-L1 mRNA expression (Figure 7A).

In subsequent experiments, TNF-α (50 ng/mL for 3 h) was used to sensitize cells (Figure 7B). The introduction of UTI89 to TNFα-sensitized MDCK cells resulted in a significant, 4.7-fold increase in PDL-1 mRNA expression compared to the un-sensitized control (*p* < 0.000, Figure 7B). Cranberry treatment, but not D-mannose, reduced the UTI89-mediated PD-L1 upregulation in TNF-α sensitized cells (3.0-fold change, *p* < 0.0001 for cranberry, and 5.5-fold change, *p* = 0.08 for D-mannose), compared to the group in which TNFα-sensitized cells were treated with UTI89 alone.

**Figure 8:**
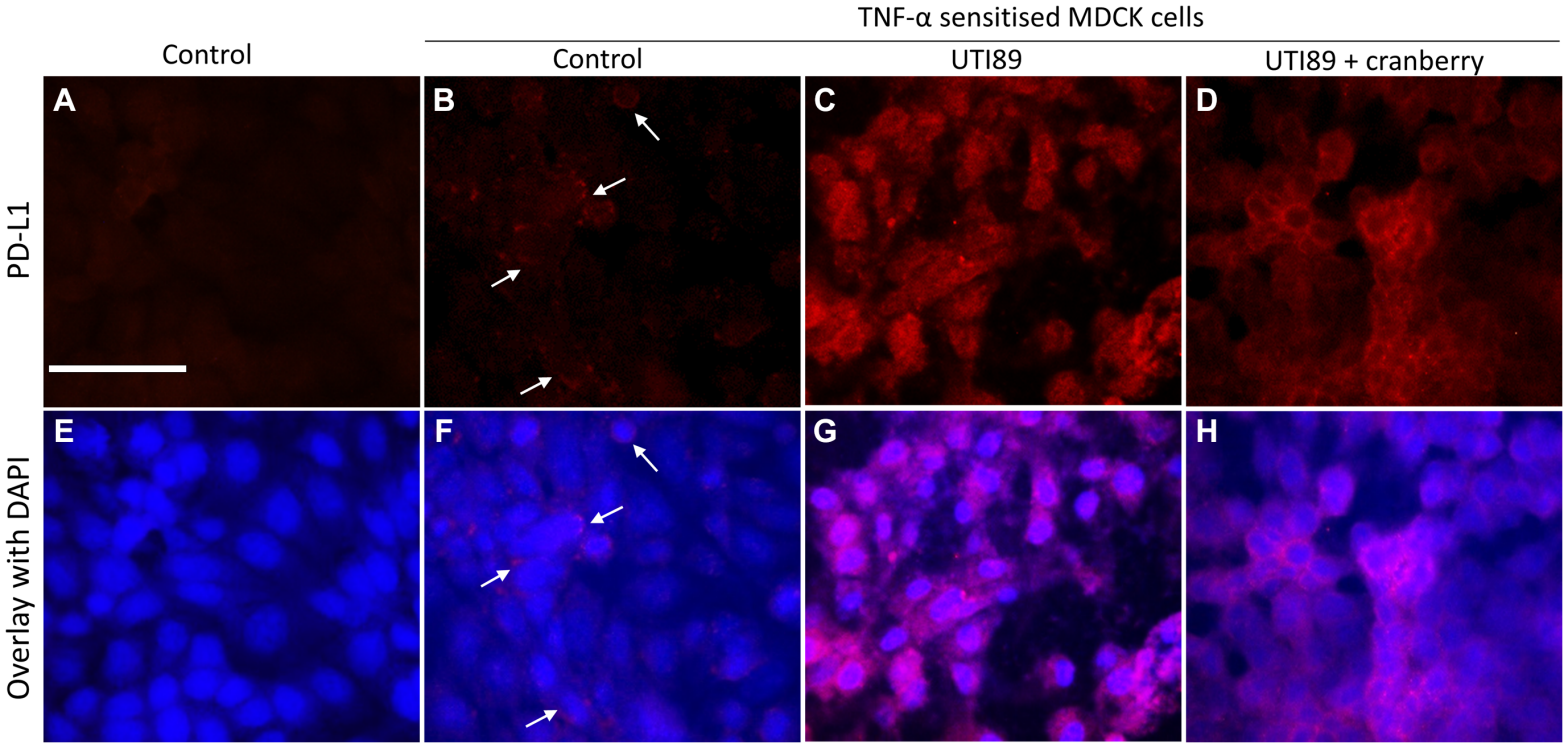
Immunocytochemistry of PD-L1 on MDCK cells. PD-L1-IR was nearly undetectable on control cells **(A,E)** and fairly visible on TNF-α treated cells [**(B,F)** indicated by white arrows]. However, the signal significantly increased following the incubation of UTI89 with TNF-α sensitized cells **(C,G)**, albeit with a slight reduction upon exposure to reduced cranberry (3 mg/mL), as shown in **(D,H)**. The images are representative of *n* = 1–2 experiments. The scale bar corresponds to 100 μm.

In control MDCK cells, PD-L1 immunoreactivity (PD-L1-IR) was almost undetectable (Figures 8A,E). When treated with TNF-α (50 ng/mL) alone, there was a mild potentiation of PD-L1-IR, resulting in a weak fluorescent signal evident on around 20% of photographed cells (Figures 8B,F). However, the introduction of UTI89 to TNF-α sensitized cells evoked a dramatic increase in PD-L1-IR, as shown by bright red immunofluorescence that completely enclosed the cytoplasmic areas of all cells (Figures 8C,G). The addition of cranberry to cells treated with both TNF-α and UTI89 partially reduced PD-L1-IR, with the immunofluorescent signal mainly localized to the edges of cells (Figures 8D,H). The effect of D-mannose on UTI89-induced increase in PD-L1-IR was minimal (data not shown).

## 4 Discussion

The standard treatment for achieving cure of UTIs is antibiotics. However, the increasing prevalence of antimicrobial resistance in uropathogens has led to increasing clinical failure rates, which has caused medical professionals to consider alternative options for treatment (Kot, 2019). Other remedies for the prevention of UTIs are needed if we are to slow the pace of antibiotic resistance development (Sihra et al., 2018). Specific non-antibiotic remedies that have been of interest in potentially providing a protective effect against UTIs clinically are cranberry, D-mannose, and ibuprofen (Konesan et al., 2022). Due to the significant clinical interest in understanding the effectiveness of these remedies, we have investigated the ways in which these three agents protect the physiological properties of the urinary tract epithelial layer.

The exposure of MDCK cells to UPEC resulted in a significant disruption and loss of the cell monolayer, attributed to its cytotoxic effect. UPEC also caused substantial disruption to the barrier function of the epithelial cell monolayers, as evidenced by decreased TEER values. This suggests that UPEC can damage epithelial cell viability and disrupt barrier integrity, leading to increased permeability of the urinary tract lining. Several studies have shown similar results regarding the effect of UPEC on the integrity of the epithelial barrier. For instance, Wood et al. (2012) demonstrated that UPEC induced significant damage to the urothelium characterized by decreased urothelial TEER when using an Ussing chamber. Another similarly designed study using rabbit bladder demonstrated that UPEC-infected urine reduced urothelial resistance, indicative of increased mucosal permeability (Tay et al., 1996). These findings are not specific to the urinary tract as a few studies have also delved into the impact of UPEC on the integrity of the gastrointestinal epithelium (Spitz et al., 1995; Shifflett et al., 2005; Bhat et al., 2019). In the gastrointestinal epithelium similar to the urinary tract, exposure to a UPEC strain led to decreased TEER (Spitz et al., 1995; Bhat et al., 2019). Hence, these findings are consistent with our experimental results, further suggesting that UPEC can cause damage to the integrity of the epithelial barrier.

Both cranberry and D-mannose are proposed to have a similar mechanism of action, which involves the non-antibiotic agent binding to the surface appendage of UPEC to prevent adhesion to the epithelium (Konesan et al., 2022). However, in the current study, we found that only cranberry, and not D-mannose, exerted a protective effect maintaining the epithelial cell viability and barrier integrity. Cranberry likely exerted this protective effect due to the presence of the active constituent of cranberry, *proanthocyanidin-A* (PAC-A), which is known to block the type P fimbriae binding domains to prevent bacterial adhesion to the *α-Gal(1–4)-β-Gal* renal glycolipid (Hansson et al., 1986; Howell et al., 2005; Spurbeck et al., 2011). These type P fimbriae are proposed to be involved in UPEC adhesion in upper urinary tract infections or pyelonephritis (Rice et al., 2005). The cell model used in these studies was MDCK cells which are derived from canine kidney therefore it is to be expected that these cells are likely to have the adhesion sites for the type P fimbriae. Conversely, D-mannose functions by inhibiting the attachment of type 1 fimbriae to uroplakin Ia, which is expressed in the urinary bladder (Ofek et al., 1982; Zhou et al., 2001). Similar to D-mannose, ibuprofen also did not elicit a preventive effect against UPEC induced damage to the epithelium. This is likely due to its mechanism of action, which involves the inhibition of prostaglandin production triggered by the inflammatory response (Wheeler et al., 2002). Unlike cranberry or D-mannose, ibuprofen does not directly bind to the surface of UPEC to block adhesion to the epithelium. Hence, this disparity may explain why ibuprofen failed to protect epithelial cell viability and integrity when challenged with UPEC *in vitro*.

Increased ROS has also been related to UTIs, as the urine of patients suffering from UTI contained malondialdehyde, a byproduct of oxidative stress (Kurutas et al., 2005). Despite this, ROS has not previously been directly quantified in UPEC-infected epithelial cells. Upregulation of ROS production is likely facilitated via bacterial LPS recognition by Toll-like receptor 4 (TLR4) receptors on the epithelial cells, which facilitates the activation of NLRP3 inflammasome mediated by ROS (Gloire et al., 2006; Mittal et al., 2014). ROS comprises reactive molecules, including oxygen and peroxides, which are increasingly produced in cells when encountering pathogenic bacteria as a defense mechanism (Li et al., 2016). ROS has antibacterial properties, used by the body’s immune response to attack invading bacteria. However, excessive ROS production can lead to oxidative stress and damage to cells and tissues in the urinary tract (Vatansever et al., 2013). Interestingly, cranberry demonstrated a reduction in ROS production against UPEC for the first 4 h. This suggests the potential role of cranberry in binding to UPEC to prevent adhesion and damage to the epithelium, thereby not triggering ROS production. However, this protective effect was not observed at 6 h. A similar result was described in Howell’s research, where the bacterial anti-adhesion activity was detectable only in urine samples of patients that consumed 240 mL of cranberry juice cocktail. The bacterial anti-adhesion activity of UPEC increased in a linear manner, peaking at 4–6 h post-consumption and then decreased after 6 h (Howell et al., 2005). This is an interesting result as most patients take cranberry once a day, and thus may explain why most clinical literature shows limited benefit. Hence, to confer protection against the progression of a UTI, consumption of cranberry may be required every 6 h. Unlike cranberry, neither D-mannose nor ibuprofen was able to lower ROS production against UPEC.

Excessive oxidative stress has been reported to initiate the inflammatory process, thus resulting in the secretion of proinflammatory cytokines such as IL-6, IL-8, and TNF-α (Lane and Mobley, 2007; Grover et al., 2011; Hussain et al., 2016). These mediators increase the activity of urinary afferent nerves, leading to urinary frequency, urgency and pain in patients with UTI (Brierley et al., 2020). The inflammatory mediator TNF-α also elevates the expression of the PD-L1 protein, which is associated with cell apoptosis (Han et al., 2020). Our results demonstrated that UPEC alone did not induce a change in PD-L1 expression in the absence of TNF-α sensitization. This is consistent with a similar finding by Lee et al. (2020) with un-sensitized colonic epithelial cells incubated with *E. coli* showing minimal PD-L1 expression compared to untreated controls. However, our study showed that in TNF-α sensitized cells UTI89 potentiated the mRNA and protein expression of PD-L1. Relevant studies suggest that this was mediated by the NF-κB pathway, activated by the ligation of TNF-α and bacterial derived LPS to TNF receptor 1 and TLR4 (Vykhovanets et al., 2012; Gowrishankar et al., 2015; Khongthong et al., 2019). Hence, our findings indicate that the NF-κB pathway, and hence cell death, is only activated when UPEC is introduced under inflammatory conditions, potentially due to TNF-α enhancing cellular recognition of bacterial derived LPS (Jaekal et al., 2007; Syed et al., 2007). Our study also revealed that cranberry, but not D-mannose partially attenuated PD-L1 mRNA and protein expression in TNF-α and UTI89-treated MDCK cells. This is consistent with our cell viability, integrity, and ROS results. One mechanism by which cranberry may have prevented UPEC induced epithelial cell death is through its inhibition of PDL-1 expression. This can potentially be explained by PAC-A in cranberry inhibiting the P fimbriae binding domains to prevent bacterial adhesion to the epithelium (Hansson et al., 1986; Howell et al., 2005; Spurbeck et al., 2011).

Taken together, our results demonstrate that cranberry is more effective than D-mannose or ibuprofen in preserving the physiological functions of the urinary tract epithelium, including cell integrity, permeability and barrier function to protect against UPEC infection. Additionally, cranberry is more efficient in preventing UPEC induced oxidative stress and inflammation induced PD-L1 expression. The optimal duration of action appears to occur at 6 h, hence clinical administration only once daily may be insufficient to confer benefit. These findings suggest the potential of cranberry to block the binding of UPEC to the epithelial surface and, consequently, prevent bacterial induced damage to the epithelial barrier.

## Data availability statement

The raw data supporting the conclusions of this article will be made available by the authors, without undue reservation.

## Author contributions

JK: Conceptualization, Data curation, Formal analysis, Investigation, Methodology, Writing – original draft, Writing – review & editing. JW: Data curation, Formal analysis, Investigation, Methodology, Writing – review & editing. KHM: Resources, Writing – review & editing. KJM: Conceptualization, Investigation, Supervision, Writing – review & editing. LL: Conceptualization, Investigation, Project administration, Resources, Supervision, Writing – review & editing.
